# Toward More Accessible Fully Automated 3D Volumetric MRI Decision Trees for the Differential Diagnosis of Multiple System Atrophy, Related Disorders, and Age-Matched Healthy Subjects

**DOI:** 10.1007/s12311-022-01472-7

**Published:** 2022-09-26

**Authors:** Jisoo Kim, Geoffrey S. Young, Ashlan S. Willett, Ariana T. Pitaro, Grace F. Crotty, Merlyne Mesidor, Kristie A. Jones, Camden Bay, Min Zhang, Mel B. Feany, Xiaoyin Xu, Lei Qin, Vikram Khurana

**Affiliations:** 1 Department of Radiology, Brigham and Women’s Hospital and Harvard Medical School, Boston, MA 02115 USA; 2 Division of Movement Disorders, Department of Neurology, Brigham and Women’s Hospital and Harvard Medical School, Boston, MA 02115 USA; 3 Department of Neurology, Massachusetts General Hospital and Harvard Medical School, Boston, MA 02114 USA; 4 Department of Pathology, Brigham and Women’s Hospital and Harvard Medical School, Boston, MA 02115 USA; 5 Ann Romney Center for Neurologic Diseases, Brigham and Women’s Hospital, Boston, MA 02115 USA; 6 Hale Building for Transformative Medicine, Brigham and Women’s Hospital, Boston, MA 02115 USA; 7 Department of Imaging, Dana-Farber Cancer Institute, Boston, MA 02115 USA

**Keywords:** Atypical parkinsonism, MSA, PSP, PD, Volumetric analysis

## Abstract

Differentiating multiple system atrophy (MSA) from related neurodegenerative movement disorders (NMD) is challenging. MRI is widely available and automated decision-tree analysis is simple, transparent, and resistant to overfitting. Using a retrospective cohort of heterogeneous clinical MRIs broadly sourced from a tertiary hospital system, we aimed to develop readily translatable and fully automated volumetric diagnostic decision-trees to facilitate early and accurate differential diagnosis of NMDs. 3DT1 MRI from 171 NMD patients (72 MSA, 49 PSP, 50 PD) and 171 matched healthy subjects were automatically segmented using Freesurfer6.0 with brainstem module. Decision trees employing substructure volumes and a novel volumetric pons-to-midbrain ratio (3D-PMR) were produced and tenfold cross-validation performed. The optimal tree separating NMD from healthy subjects selected cerebellar white matter, thalamus, putamen, striatum, and midbrain volumes as nodes. Its sensitivity was 84%, specificity 94%, accuracy 84%, and kappa 0.69 in cross-validation. The optimal tree restricted to NMD patients selected 3D-PMR, thalamus, superior cerebellar peduncle (SCP), midbrain, pons, and putamen as nodes. It yielded sensitivities/specificities of 94/84% for MSA, 72/96% for PSP, and 73/92% PD, with 79% accuracy and 0.62 kappa. There was correct classification of 16/17 MSA, 5/8 PSP, 6/8 PD autopsy-confirmed patients, and 6/8 MRIs that preceded motor symptom onset. Fully automated decision trees utilizing volumetric MRI data distinguished NMD patients from healthy subjects and MSA from other NMDs with promising accuracy, including autopsy-confirmed and pre-symptomatic subsets. Our open-source methodology is well-suited for widespread clinical translation. Assessment in even more heterogeneous retrospective and prospective cohorts is indicated.

**Supplementary Information:**

The online version contains supplementary material available at 10.1007/s12311-022-01472-7.

## Introduction

Early and precise diagnosis of neurodegenerative diseases is becoming increasingly pressing with disease-modifying therapies on the horizon and multiple clinical trials underway. Multiple system atrophy (MSA) — a neurodegenerative movement disorder (NMD) presenting with predominantly cerebellar (MSAc) or parkinsonian (MSAp) features — is an attractive target for therapeutics because of its relatively rapid course, orphan status, and biological connection to Parkinson’s disease (PD). Accurate differential diagnosis of MSA, particularly in its early stage, from other parkinsonian and ataxic NMDs is likely to be important for successful clinical trials. This differential dignosis, however, remains a challenge for subspecialist and generalist neurologists alike. In many cases, years of suffering, uncertainty, health care cost, and lost research opportunities accrue between initial presentation and diagnosis by a movement disorder subspecialist [1].

To increase rates of accurate early diagnosis and subspecialty referral, new diagnostic tools translatable to widespread community use are needed [2]. Mimics of MSA among so-called sporadic disorders (without known genetic cause) include Parkinson’s disease (PD) that mimics MSAp. Progressive supranuclear palsy (PSP) can also present with parkinsonism or ataxia-predominant (PSPc) symptomatology and can thus mimic MSAp or MSAc, respectively [3, 4].

Brain MRI with 3D T1-weighted images (3DT1) is now widely available, relatively inexpensive, and commonly used in initial community-based workup of neurological and psychiatric conditions. Successful widespread community translation of 3DT1 volumetrics could facilitate early identification and subspecialty referral of MSA patients and improve reproducibility of early diagnosis. But equally, this methodology could assist movement disorder sub-specialists seeking objective data for differential diagnosis of early-stage patients and their triage into appropriate clinical trials.

Extensive previous work has established a number of 1D and 2D measurements as potentially useful for the diagnosis of PSP and MSA [5–10]. However, 1D and 2D measurements are significantly affected by differences in acquisition (including slice thickness, slice registration, and slice angulation), as well as operator-dependent differences in measurement technique. Thus, successful use of these measures requires meticulous MRI acquisition and measurement, including standardized protocols and expert operators. These conditions are hard to meet outside leading research centers.

Important recent work has demonstrated the potential of atlas-based automated segmentation of 3DT1 data to improve reproducibility. In particular, Messina et al. [11] used automatic volumetric segmentation of cerebellum, thalamus, putamen, pallidum, hippocampus, and brainstem to demonstrate different patterns of brain atrophy in MSAp, PSP, and PD. Krismer et al. [12] demonstrated differences between putamen and cerebellum volumes in MSA and PD. Barbagallo et al. [13] analyzed multiple MRI parameters including volumetric measurements of the nigro-striatal pathway.

Two important studies that deserve special mention provided early proof-of-principle support for use of automated volumetric analysis in differential diagnosis of MSA from other NMDs [14, 15]. Scherfler et al. [14] reported an algorithm differentiating carefully characterized MSA, PSP, and PD patients. Their approach utilized open-source Freesurfer MRI atlas software and was based on semi-automated processing of volumes of midbrain, putamen, and cerebellar gray matter with manual segmentation of brainstem structures. In that study, there was meticulous curation of early-stage patients of defined ages and disease durations. Presynaptic nigrostriatal dopaminergic dysfunction was confirmed by dopamine transporter SPECT and 18F-dopa PET. Moreover, the “decision tree” analysis method used in that study was appealing because it is resistant to data overfitting and is easily followed, entailing a straightforward step-by-step decision-making process that involves binary questions related to specific brain volumes [16]. Nevertheless, the partial automation of the approach in conjunction with the rigorous workup of patients in this study leaves unanswered the question of how broadly applicable the methodology is to community settings. In these settings, patient populations are more loosely curated and fully automated algorithms are required.

In a contemporaneous study, Huppertz et al. [15] applied fully automated volumetric MRI analysis to a large cohort of MSAc, MSAp, PSP, and PD patients. They employed a different atlas-based software and support vector machine (SVM) analysis. SVM is an early high-dimensional multivariate supervised machine-learning approach. The approach utilizes one or more high-dimensional decision “hyper-planes” to classify individuals within a multi-variate dataset. SVM and newer deep learning approaches that are replacing it offer greater discriminatory power than decision trees or multivariate logistic regressions, but are less intuitive to understand and interpret. They are also prone to overfitting in small dataset. They have thus proved difficult to generalize. For this reason, in the current study, we opted for the simpler decision tree approach as it offers greater potential for successful widespread clinical translation in the community but, like the Huppertz study, we opted for a fully automated approach [15].

We employed a two-step approach toward broader translation by producing 2 complementary decision trees — first a decision tree to guide differentiation of NMD patients from healthy subjects, and second a separate decision tree to assist differentiation among NMDs. We included brainstem sub-segmentation [17] using freely available open-source volumetric software and recursive partitioning with decision tree analysis [18]. We used a more recent software version than the Scherfler study and careful input-volume selections to address known sources of segmentation error [19–21]. We also introduced a novel parameter, the pons-to-midbrain volume ratio (3D-PMR). Notably, we applied and internally validated this approach in a retrospective cohort comprising all ascertainable MSAp and MSAc patients in our hospital network who had undergone 3DT1 MRI at any timepoint in their disease course, an approximately matched number of PD and PSP patients from the same network, and a healthy subject dataset matched for age and legal-sex. The technically and clinically heterogeneous retrospective dataset drawn from our large tertiary hospital system was selected to test for wider translatability of our approach. Moreover, we tested decision-tree performance in a small but important group of patients with autopsy-confirmed diagnoses and in patients with MRI before motor symptom onset, two subsets of patients not evaluated in prior studies.

We have found that our approach does in fact distinguish NMD patients from healthy subjects and also sub-classifies NMDs with promising sensitivity and specificity, including autopsy-confirmed and pre-symptomatic subsets. These data suggest that our open-source methodology may be well-suited for widespread clinical translation and warrants testing in larger, prospective cohorts in different clinical settings. We hope the current study provides an important step in clinical translation of volumetric MRI for the differential diagnosis of MSA and related disorders.

## Methods

Our institutional review board approved this retrospective medical records study with a waiver of informed consent.

### Cohorts

**Fig. 1:**
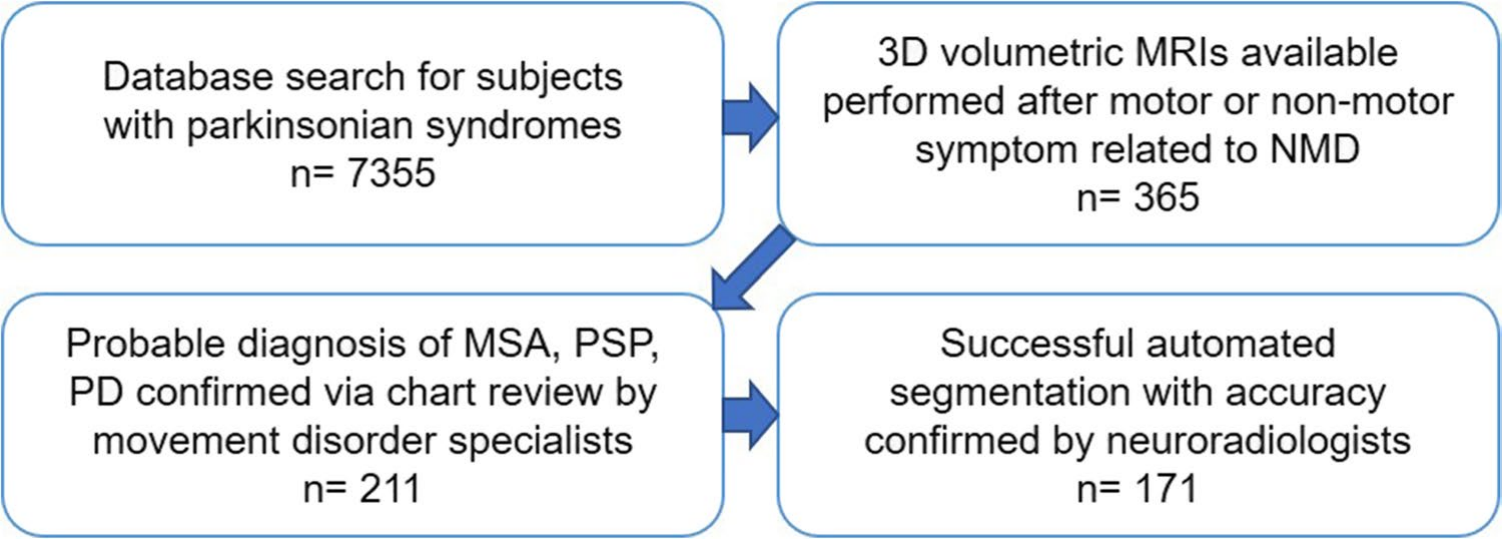
Flow chart of inclusion criteria of our patient cohort

We identified all subjects with MSA and approximately matched number of subjects with other NMD within our hospital network, using both a research tool embedded in the hospital electronic medical record system and an automated research query tool developed at our institution. The search terms used were “Parkinson Disease,” “Multiple System Atrophy,” “Progressive Supranuclear Palsy,” and “Parkinsonian.” Patients were included who (1) met “probable” or “pathologically-confirmed” consensus criteria for MSA [22], PSP [23], or PD [24], and (2) had spoiled gradient echo 3DT1 brain MRI data of acceptable quality (Fig. 1). MRIs performed for unrelated reasons prior to any symptom onset related to NMD were excluded. If multiple MRIs were performed for a single patient, the earliest MRI was chosen among those that were performed after initial presenting symptom onset. In most cases, the initial NMD presenting symptom was a motor symptom, but in an informative minority of patients was non-motor, offering an opportunity for us to analyze the effectiveness of our method before motor symptom onset (which would be ideal for early diagnosis). If motor symptom was present at the time of MRI, predominant symptom was recorded as ataxia, parkinsonism, or both. Medical records of the included patients were assembled by clinical research coordinators (AW, MM) and reviewed by neurologists GC (clinical movement disorders subspecialist with 8 + years of training) and VK (faculty movement disorders subspecialist with 12 + years of experience). Demographic data collected include birth date, legal sex, year of motor symptom onset, age at MRI, predominant motor symptom at the time of MRI if motor symptom was present, and duration of clinical follow-up following motor symptom onset (Table 1).

**Table 1:** Patient demographics of our patient cohort and legal sex, age-matched healthy subject cohort. For 6 patients, MRI date preceded motor symptom onset (1 MSAc, 1 MSAp, 4 PSP)

Cohort	Diagnosis (probable/autopsy confirmed)	Legal sex (F/M)	Age at MRI (yrs)	Age at motor symptom onset (yrs)	Disease duration to MRI (yrs)	Motor symptom at MRI (ataxia/parkinsonism/both)	Clinical follow-up since motor symptom onset (yrs)
MSA	54/18	31/41	62.8 ± 8.0	59.6 ± 7.8	3.2 ± 2.5 (− 0.4–11.7)	37/14/19	7.2 ± 3.2
*MSAc*	*36/14*	*21/29*	*62.5* ± *8.1*	*58.9* ± *8.3*	*3.3* ± *2.4 (0*–*11.7)*	*37/0/12*	*7.5* ± *3.3*
*MSAp*	*18/4*	*10/12*	*64.3* ± *7.9*	*60.3* ± *7.3*	*2.7* ± *2.6 (*− *0.4*–*10.0)*	*0/14/7*	*5.9* ± *2.6*
PD	41/8	16/33	67.2 ± 9.2	60.9 ± 10.4	6.7 ± 4.6 (0–15.8)	0/47/0	12.3 ± 5.6
PSP	42/8	27/23	70.6 ± 7.7	67.72 ± 8.0	2.9 ± 3.0 (− 1.4–15.9)	4/40/2	6.3 ± 2.7
Total	137/34	74/97	66.3 ± 8.9	61.95 ± 8.5	4.1 ± 3.7 (− 4.1–15.9)	41/101/21	8.3 ± 4.6
Healthy subject	N/A	74/97	66.8 ± 8.9	N/A	N/A	N/A	N/A

We adopted multiple measures to improve upon the robustness of key prior studies (Table 2 [14, 15]). Notable strengths of our study are a relatively heterogeneous patient cohort that more closely mimics the community setting, one-on-one matching of healthy subjects, inclusion of autopsy-confirmed patients, full automation with a more recent version of open-source software that exhibits reduced segmentation error, use of an unbiased and interpretable statistical model, and subset-analysis of MRIs preceding motor symptom onset. In addition, we used a larger set of healthy subjects: we selected a total of 171 healthy subjects with MRI matching our disease cohorts one-on-one by age and legal sex. These were obtained from OASIS-3 (Open Access Series of Imaging Studies), a clinical and imaging database of 1098 participants, ranging from 42 to 95 years and including 605 cognitively normal adults and 493 patients with cognitive decline [25]. Using the database’s clinical records, we excluded those with neurodegenerative disease or a movement disorder based on one or multiple records of Alzheimer’s Disease Research Center clinical data, Unified Parkinson’s Disease Rating Scale (UPDRS) Part 2 scorings, physical neurological examination findings, and medical/health histories. For the healthy subjects we selected, we also noted whether the participant had a history of alcohol substance use disorder, mood disorders, cerebrovascular disease, B12 deficiency, thyroid disease, and among other potential confounding factors.

**Table 2:** Comparison with key prior studies in the field [14, 15]

	Present study	Scherfler et al. (2016)	Huppertz et al. (2016)
Training cohort size	72 MSA (50 MSAc, 22 MSAp), 49 PD, 50 PSP	26 MSA, 20 PSP, 26 PD	81 MSA (21 MSAc, 60 MSAp), 106 PSP, 204 PD
Testing cohort size	Same as training set	14 MSA, 10 PSP, 14 PD	Same as training set
Disease duration at time of MRI (years)	Mean 2.7–6.7 / SD 2.4–4.6 (− 4.1–15.9)	Mean 2.5–3.3 / SD 1.2–2.2 (all < 6)	Mean 3.2–6.5 / SD 0.2–0.5 (0.3–23.4)
Age at MRI (years)	47–92	50–75	35–88
Supportive testing	-	dopamine SPECT or [18F]-dopa PET	-
Healthy subjects used?	Yes; age, legal sex matched (*n* = 171)	Yes; age matched (*n* = 41)	Yes (*n* = 73)
Pathologically confirmed cases?	Yes; MSA (14 MSAc, 4 MSAp), 8 PSP, 8 PD	No	No
Fully automated?	Yes	Semi-automated (automated brain and whole brainstem, manual brainstem sub-segmentation)	Yes
3D MRI processing software (release date)	Freesurfer v6.0 (2017)	Freesurfer v5.1 (2011)	Matlab SPM12 (2014)
Model	Decision tree	Decision Tree	Support vector machine
Model generation software	Rpart v4.1–15 (R v3.6.1)	C4.5 (WEKA 3.6)	LIBSVM v3.20
Validation	tenfold 10-repeat cross validation	33–35% hold-out validation	tenfold cross validation
Exploration with MRIs preceding motor symptom onset	Yes	No	No

### Autopsy-Confirmation

Results of postmortem examinations performed at our institution according to standard Alzheimer Disease Research Center protocols were retrieved from the medical record. Briefly, brain samples were bisected longitudinally. One half was coronally sectioned and rapidly frozen, and the other half was fixed in 10% (vol/vol) neutral buffered formalin and then sectioned. Histological evaluation of multiple brain regions included staining with LFB, H&E, and immunohistochemical analysis for α-synuclein, β-amyloid, and phosphorylated tau. Histopathologic diagnosis was confirmed according to standard criteria [26–28]. A clinical MSA diagnosis (whether MSAp or MSAc) was confirmed neuropathologically if defined consensus criteria were met [29], including the presence of alpha-synuclein-rich glial cytoplasmic inclusions.

### Volumetric Image Analysis

**Fig. 2:**
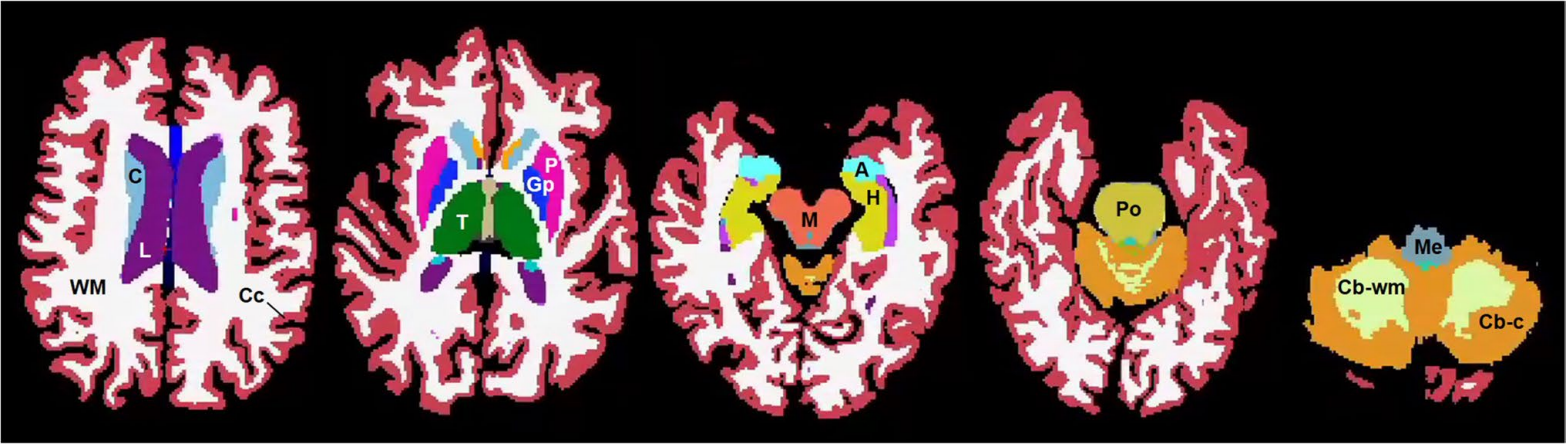
Example of fully automated segmentation of the brain and brainstem produced by Freesurfer 6.0 and its brainstem module. Key structures are labeled as following from left to right; WM, cerebral white matter; C, caudate; L, lateral ventricle; Cc, cerebral cortex; T, thalamus; Gp, glospus pallidus; P, putamen; M, midbrain; A, amygdala; H, hippocampus; Po, pons; Cb-wm, cerebellar white matter; Me, medulla; Cb-c, cerebellar cortex

Fully automated volumetric analysis was performed running on a high-performance research computing cluster, using Freesurfer v6.0 which is documented and freely available for download online (http://surfer.nmr.mgh.harvard.edu/) [30]. The Freesurfer Brainstem Substructures module was applied to automate the segmentation of the pons, midbrain, medulla, and SCP [17]. Each segmented MRI was examined for accuracy by a neuroradiologist (JK) and selected images were reviewed by a senior neuroradiologist (GY) on Freeview, a tool developed by Freesurfer developers to visualize Freesurfer output (Fig. 2).

### Statistical Analysis

A biostatistician (CB) guided and reviewed all statistical analysis. We used a Classification and Regression Tree (CART) algorithm using rpart v4.1–15 in R (v3.6.1) which performs unbiased recursive partitioning, yielding automated decision trees. Decision trees offer superior interpretability and clinical translatability [18] as exemplified through prior literature (Table 2). Ratio of pons-to-midbrain volume (3D-PMR) and volumes of midbrain, pons, SCP, caudate, putamen, striatum, thalamus, pallidum, cerebellar white matter, hippocampus, amygdala, lateral ventricles, third ventricle, fourth ventricle, and choroid plexus were used as input variables. All of these structures were used since a combination of these variables may provide better prediction and accuracy, as reported with previous MRI biomarkers such as the Magnetic Resonance Parkinsonism Index and its 2.0 counterpart [7, 8]. 3D-PMR drew inspiration from the midbrain-to-pons area ratio [6, 10]. In prior literature, the ratio of midbrain area to pons area has been shown to distinguish PSP from PD, MSA, and healthy subjects. We hypothesized that a fully automated volumetric counterpart of this ratio would be a robust measure to distinguish NMDs. Striatum volume was derived by adding the volumes of caudate, putamen, and nucleus accumbens. Cerebellar gray matter, cerebral cortex, cerebral white matter, and corpus callosum were excluded because the segmentations were deemed inconsistent and/or inaccurate based on prior literature and expert review of our data [21]. Other Freesurfer outputs including the optic chiasm and white matter hyperintensities were excluded because, based on prior literature, they were deemed unlikely to be closely associated with the pathophysiology of the relevant NMD and thus more likely to contribute to spurious correlation within the model (i.e., overfitting) than correct discrimination of the NMD. The first decision tree was made to differentiate healthy subjects from diseased, and a second to differentiate among NMDs. Our models were validated by tenfold, 10-repeat cross-validation with the caret package in R. Finally, we also explored one of the most vexing aspects of the differential diagnosis with our approach, namely the differential diagnosis of only those who presented with parkinsonism.

## Results

In our cohort, disease groups had no statistically significant differences in distribution of legal sex (chi-square test) or age at motor symptom onset (Mann–Whitney test). The MSA group had a lower age at the time of their MRI and the PD group had a longer time between motor symptom onset and MRI. Review of documented presenting motor symptoms revealed that at the time of initial MRI, 12/50 (24%) MSAc, 7/22 (32%) MSAp, and 2/50 (4%) PSP patients exhibited both ataxia and parkinsonism, and 4/50 (8%) PSP patients had predominantly ataxia (Table 1). For 6 patients, MRI date preceded motor symptom onset but were performed after initial presenting symptoms related to NMD such as orthostasis, psychomotor slowing, flat affect, memory changes, and falls (1 MSAc, 1 MSAp, 4 PSP). For 2 patients, additional MRIs prior to symptoms related to NMD were available (2 PD). These were performed for transient aphasia and seizure of unknown etiology for one patient and vision changes for another patient which were eventually thought to be due to glaucoma.

**Fig. 3:**
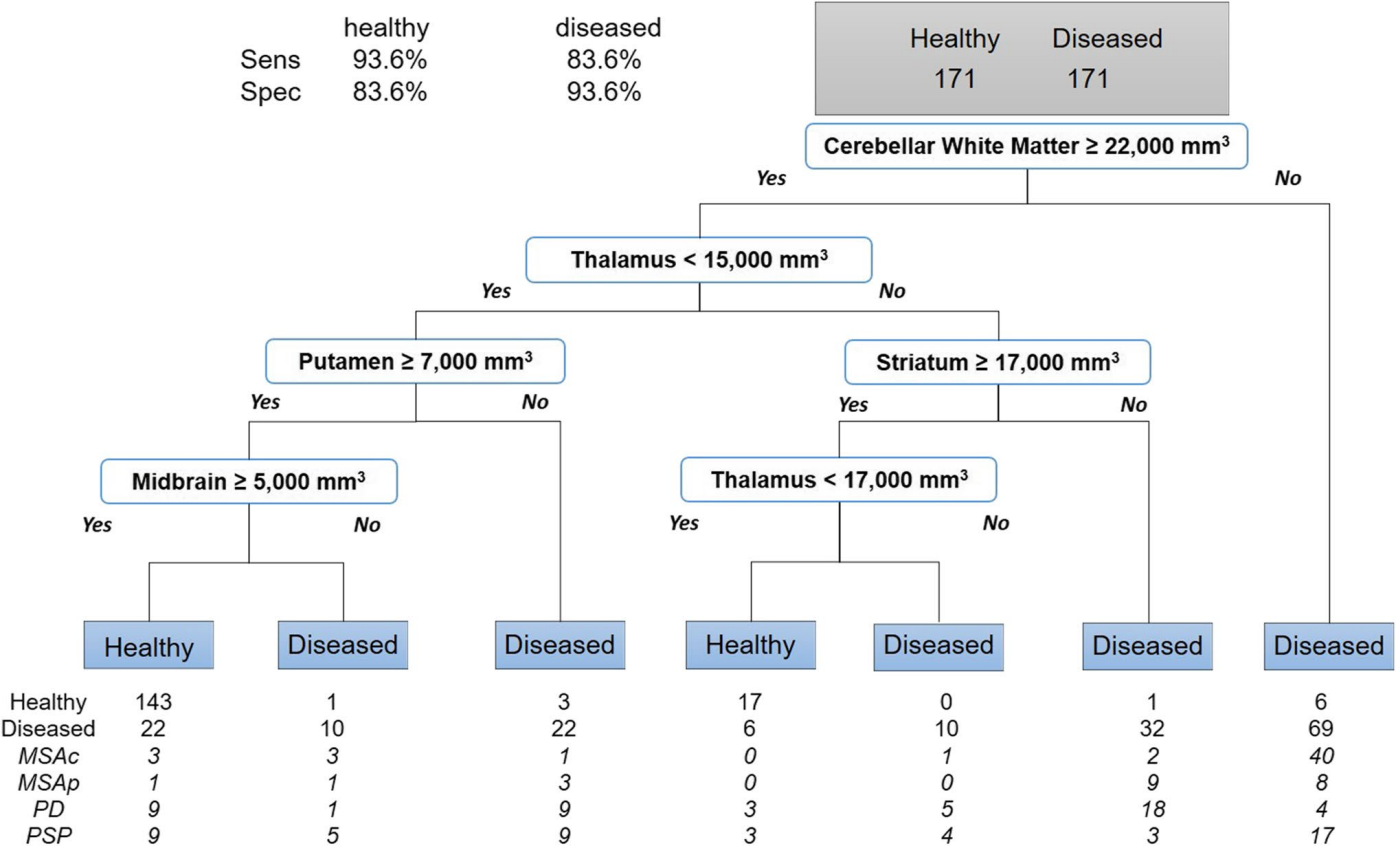
Screening decision tree — healthy vs NMD. (3D-PMR, pons-to-midbrain volume ratio; MSAc/p, multiple system atrophy-cerebellar/Parkinsonian subtypes; PD, Parkinson disease; PSP, progressive supranuclear palsy)

The most high-level decision-tree created was a screening tree to differentiate healthy subjects from NMD patients. Through automated, unbiased optimization for the highest accuracy, this algorithm selected cerebellar white matter as its first node with a value smaller than 22,000 mm^3^ being classified as diseased (Fig. 3). Then, it selected thalamus, putamen, striatum, and midbrain volumes as nodes (Fig. 3). The resulting optimal decision-tree demonstrated sensitivity of 83.6%, specificity of 93.6%, and positive predictive value of 92.9% for the diseased group with 84.4% accuracy and 0.69 kappa from cross-validation. This tree correctly classified 17/18 MSA, 7/8 PD, and 5/8 PSP autopsy-confirmed patients as diseased (85.3% accuracy). All autopsy results confirmed the clinical diagnosis prior to death except for 1 PD and 2 PSP patients whose clinical diagnoses were unclear. In these cases, we adopted the diagnoses revealed from autopsy. Of the patients who had MRIs before their motor symptom onset, 1/1 MSAc, 1/1 MSAp, 2/2 PD, and 3/4 PSP patients were correctly classified as diseased, yielding 87.5% accuracy.

**Fig. 4:**
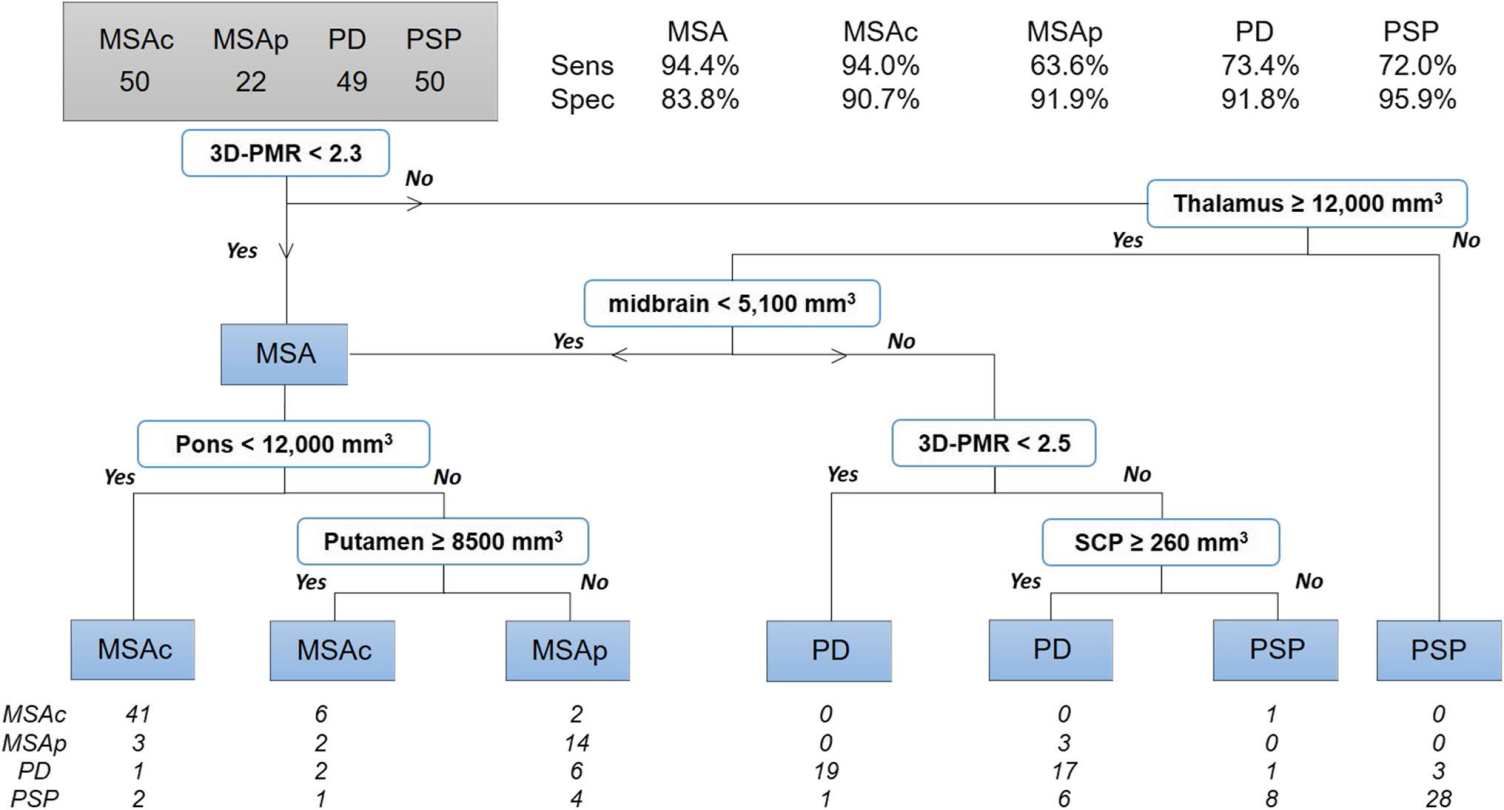
Comprehensive differential diagnosis tree — MSA vs MSAc vs MSAp vs PD vs PSP. (3D-PMR, pons-to-midbrain volume ratio; SCP, superior cerebellar peduncle; MSAc/p, multiple system atrophy-cerebellar/Parkinsonian subtypes; PD, Parkinson disease; PSP, progressive supranuclear palsy)

Next, a comprehensive tree was created to subclassify patients within the NMD group. This algorithm selected our novel biomarker, 3D-PMR, as its first node with a value smaller than or equal to 2.3 being classified as MSA (Fig. 4). This biomarker alone differentiated MSA from other diseases. The algorithm then selected thalamus, midbrain, again 3D-PMR, and SCP as its nodes (Fig. 4). This yielded sensitivity and specificity of 94.4 and 83.8% for MSA, 73.4 and 91.8% for PD, and 72.0 and 95.9% for PSP.

We attempted a variant of this tree to further sub-divide patients with MSA into cerebellar and parkinsonian subtypes, although we were under-powered to do so with the paucity of MSA patients. This analysis (lower left of Fig. 4) demonstrated sensitivity and specificity of 94.0 and 90.9% for MSAc, 63.6 and 91.9% for MSAp. Cross-validation revealed 78.5% accuracy and 0.62 kappa.

In subset analyses, our NMD decision tree correctly classified 17/18 MSA, 6/8 PD, and 5/8 PSP autopsy-confirmed patients constituting 82.4% accuracy. Of the 18 autopsy-confirmed MSA patients, 14/14 MSAc and 3/4 MSAp patients were correctly classified, with one MSAp patient mis-classified as MSAc. In a second subset analysis, of MRIs obtained before motor symptom onset, 1/1 MSAc, 1/1 MSAp, 2/2 PD, and 3/4 PSP patients were correctly classified, yielding 87.5% accuracy. One PSP patient was misclassified as PD.

For differentiating NMDs in patients with parkinsonism at the time of MRI with or without ataxia, decision tree algorithm selected SCP, 3D-PMR, pons, and 3rd and 4th ventricles, resulting in sensitivity and specificity ranging 67–89% and 79–99%, respectively, for MSAc, MSAp, PD, and PSP (Supplementary Fig. 1).

## Discussion

One of the major objectives of the current study was to develop an accessible approach to the differential diagnosis of MSA from other clinically related NMD diagnoses. We envision that our approach might be adapted to a community setting where 3D MRI is readily available. To facilitate this adaptation, we focused on a fully automated approach that employed open-source software and a statistical method that is unbiased, intuitive, and widely translatable (i.e., decision tree). Moreover, we purposely included patients from across a large tertiary level hospital center to create a more clinically heterogeneous cohort that had been subjected to a range of MRI scanners and methodologies. We reasoned such a cohort would better mimic a community-based population.

The post-hoc analysis of presenting symptom at the time of initial MRI revealed mixed or atypical presenting symptoms in 24% MSAc, 32% MSAp, and 12% PSP patients (Table 1). Taken together with the 4% of the cohort with non-motor symptoms only at time of initial MRI, these findings illustrate that early in the course of NMD, differentiation on clinical grounds can be quite difficult, underscoring the importance of creating objective methods to assist early differential diagnosis of NMD patients that are independent of clinical differentiation between ataxic and parkinsonian presentation.

For differentiating NMD, a novel measure, the 3D-PMR, proved valuable. Drawing inspiration from the 2D literature in which midbrain and pons areas on mid-sagittal plane were utilized on their own, as a ratio, or as part of a formula, we developed this measure as a ratio of pons to midbrain volumes [5–10]. Interestingly, 3D-PMR was not only the key upstream node for distinguishing MSA from other NMDs likely related to pontine atrophy in MSA but also useful in distinguishing PSP and PD, likely due to midbrain atrophy observed in PSP (Fig. 4). MSA patients who did not meet the criteria of low 3D-PMR were correctly classified further downstream in the decision tree by excluding thalamic atrophy and with suggestion of midbrain atrophy, which correlates with findings from prior literature of midbrain atrophy in MSAp [31]. Overall, the presence of 3D-PMR at multiple points of the tree suggests that it can be served as a gradient biomarker for differentiating multiple NMDs, rather than a binary decision maker.

Notably, our two decision-tree approaches (Figs. 3 and 4, respectively) were intended for two distinct purposes. Our initial approach — to distinguish our NMD from healthy subjects (Fig. 3) — was designed to simulate the type of initial filter one might employ among community clinicians and imagers to identify NMD patients among the wide variety of patients with various pathologic and functional disorders potentially mimicking NMDs such as essential tremor, Wernicke’s syndrome, communicating hydrocephalus, and spinal disorders among others. The screening decision-tree produced a high sensitivity/specificity of 84/94% and cross-validated accuracy of 84.4% in our relatively technically heterogeneous cohort. This supports the hypothesis that our approach may be able to help community physicians facing cohorts of patients with movement issues of various etiologies to identify NMD patients for specialist referral. The tree may be useful even before motor symptom onset as it showed an even higher accuracy of 88% for predicting the presence of a NMD in patients with MRI before motor symptom onset. Both of these results will need to be validated on a larger and even more heterogeneous cohorts incorporating patients with various movement-related impairments unrelated to NMDs.

This screening tree (Fig. 3) identified lower volumes — likely from atrophy — of the cerebellar white matter, striatum, putamen, and midbrain in NMD patients compared to healthy subjects. For thalamus, however, large volume suggested NMD, which may be due to thalamic enlargement observed in PD [32]. The consistency of these results with previous literature and with the generally accepted neuropathology and pathophysiology of NMDs is very encouraging for clinical translation because it suggests that the correlations embedded in this decision-tree method are biologically relevant and thus potentially generalizable.

The second decision tree (Fig. 4) was designed to assist neurology specialists and movement disorder sub-specialists in differentiating among the various NMDs, once patients with unrelated movement issues have been excluded. This tree provided more granular understanding of the specific volumetric differences that had emerged in our first decision tree: the small pons volumes were driven by pontine atrophy in MSAc and small striatum volumes by putaminal atrophy in MSAp. Thalamic atrophy suggested PSP and distinguished from PD as well as MSA, a finding, as noted above, that is consistent with previously reported enlarged thalami in PD in addition to previously reported thalamic atrophy in PSP [32, 33]. SCP atrophy suggested PSP as observed in prior literature [34, 35].

The second tree (Fig. 4) demonstrated specificity of 83.8–95.9% and sensitivity of 72.0–94.4% for pairwise comparison of MSA vs PD vs PSP. Further branching of this tree designed to differentiate MSA into MSAp and MSAc was also attempted, which differentiated MSAp with high specificity (91.9%) but a low sensitivity of 63.6%. We presume this less effective performance resulted from our much smaller sample size for MSAp patients. Additionally, overlapping phenotypes between MSAp and MSAc as well as MSAp and PD patients likely contribute to the lower sensitivity of MSAp group, which again emphasize the difficutly in differential diagnosis [35, 36]. Consistent with this possibility, 5 of the 8 incorrectly classified MSAp patients were classified as MSAc and 3 as PD in the second decision tree (Fig. 4). Furthermore, for our autopsy-proven MSAp cases, 1/4 was incorrectly classified by the decision tree as MSAc. The high performance in patients with autopsy-confirmed diagnoses (82%) and MRIs before motor symptom onset (88%) is notable. While the numbers are small, this result suggests that our approach may prove robust in identification of patients with NMDs, particularly MSA, early in the disease course. This would be highly desirable, not only to decrease patient suffering and healthcare costs but also to triage patients for therapeutic trials at the early stages where disease-modifying interventions are most likely to work. This will by synergistic with the efforts for early and accurate diagnosis, for example through CSF analysis for α-synucleinopathies or skin biopsy for PD (*also see accompanying submission*, *Ndayisaba*, *Pitaro*, *Willett *et al*. The Cerebellum*, *this issue*) [37, 38].

Finally, our supplementary decision tree (Supplementary Fig. 1) generated with data from a sub-group of patients who had parkinsonism resulted in SCP as its first node with atrophy suggesting MSA, PD, or PSP. Without SCP atrophy, a larger 4th ventricular size, which is an indirect measure of cerebellar atrophy, suggested MSAp rather than PD. Smaller 3D-PMR suggested MSA, among which pontine atrophy pointed towards MSAc. Larger 3D-PMR, along with larger 3rd ventricular size, which is an indirect measure of thalamic atrophy, suggested PSP. Although underpowered, this tree demonstrated relatively high specificity (79–99%) which suggests that with additional data, this approach can assist with differentiation of those presenting with parkinsonism, which is among the most challenging for differential diagnosis in NMDs.

Although our sample size is relatively small, especially in the MSAp cohort, this is still one of the largest comprehensive cohorts yet published, consistent with the rarity of atypical parkinsonism-ataxia spectrum disorders (Table 2). Future studies with still larger cohorts and multi-center cohorts are needed. Heterogeneity of the disease cohort, such as the older age of the PSP and PD groups, is a potential source of bias, although the inclusion of age and sex-matched healthy subjects should somewhat mitigate against this. Among the sources of this heterogeneity, the older age of the PSP and PD groups likely reflects later average symptom onset in these diseases compared to MSA. This may have degraded the performance of our classifiers. However, because this age distribution should more closely simulate a real community-clinic populations, the strong overall performance of our decision trees suggests that our method will continue to perform well in prospective community application. The longer interval from symptom onset to MRI in the PD group is another limitation that may mirror future clinical use because of the slower progression of symptoms in PD. Interestingly, the two PD patients with earlier MRIs available preformed before any symptom onset were correctly classified as PD, supporting the notion than the classifier may perform better in the critical early detection task. Prior to prospective clinical use, studies with longitudinal analysis of patients with MRIs at multiple time points will be needed to understand the effect of symptom duration on MRI volumetric diagnosis of NMDs [17]. Variability in the timing of MRI in relation to symptom onset is another limitation, inherent to a retrospective study. While this likely decreases the overall measured sensitivity and specificity of the decision trees, it reflects the variability in practice patterns among providers ordering MRIs. As such, the reports’ sensitivities and specificities are likely to approximate those that will be encountered in clinical use of the trees.

As noted, we included only 3D-PMR and volumes of midbrain, pons, SCP, caudate, putamen, striatum, thalamus, pallidum, cerebellar white matter, hippocampus, amygdala, lateral ventricles, third ventricle, fourth ventricle, and choroid plexus in the input set when performing the decision tree analysis. This excluded a number of other structures that are known not to be involved in the NMD of interest, or for which low segmentation reliability is either known from prior literature or was identified in our study. In particular, we excluded cerebellar gray matter, cerebral cortex and white matter, and corpus callosum volumes which might otherwise have been of interest, because of known limitations of Freesurfer v6.0 accuracy for cerebellar gray matter and inaccurate segmentation of the supratentorial volumes in a number of patients on visual review. We also eliminated nucleus accumbens, a relevant structure of interest, as an independent volume, merging it with putamen and caudate in our striatum volume, because the accumbens boundaries cannot be discerned readily with T1-weighted imaging. Notably, we used a more advanced version of Freesurfer, v6.0 (Table 2), which was a major upgrade from the previous v5.3 with improvements in registration, segmentation, and classification. In particular, this version has improved putamen segmentation and automatic brainstem sub-segmentation module [17, 20, 39]. For these reasons, the resulting classifier may prove to be more generalizable than earlier approaches.

Our study illustrates the major advantage of decision-tree: it is a more interpretable and transparent statistical method compared to more complex SVM and deep learning approaches that can appear as a “black box” to the broader clinical community. The ability to understand the basis for individual patient classifications is one of the main advantages of this approach. Put another way, it is a simple, interpretable, and transparent statistical analysis and modeling technique. Such methods reduce the likelihood of data overfitting, classifier performance drift, and performance degradation in application to external datasets while at the same time engender physician acceptance and trust in the method. Bayes’ theorem dictates that the positive predictive value of any test depends heavily on the pre-test prevalence of disease in the test population [40]. Therefore, accuracy of any classifier is expected to decrease when it is applied to less carefully selected populations, and careful stepwise validation in progressively more heterogeneous populations is required to translate diagnostic methods developed in carefully characterized patient cohorts to broader use. Validation across multiple independent datasets is warranted for this technology to become widely accepted.

## Conclusion

We have demonstrated that fully automated diagnostic decision-trees can perform well in differentiating NMDs from healthy subjects and in the differential diagnosis of MSA from other NMD. We applied the methodology in technically heterogeneous clinical 3DT1 brain MRI data on a relatively loosely selected retrospective patient cohort. Validation, albeit with limited numbers, in pre-symptomatic and autopsy-proven cases was promising. Our findings justify additional work toward widespread community translation aiming to assist in earlier, more accurate detection of NMD patients so as to allow earlier appropriate referral to subspecialty movement disorder centers, reduce patient suffering and medical costs, and facilitate the early triage of patients to therapeutic intervention trials. The dataset curated from our large tertiary healthcare system with an age and legal sex-matched healthy subject cohort will be valuable for future collaborative efforts, as the international MSA community builds the clinically heterogeneous admixed cohorts that are needed for external validation and eventual translation to diverse community settings. In the subspecialty setting, such volumetric approaches may prove synergistic with functional data from diffusion-weighted imaging, diffusion tensor imaging, or other advanced MRI approaches that are being applied to the differential diagnosis of MSA and related disorders.

## Supplementary Information

Below is the link to the electronic supplementary material.

Supplementary Fig.1 Parkinsonism differential diagnosis tree – MSAc vs MSAp vs PD vs PSP who had parkinsonism with or without ataxia at the time of MRI. (3D-PMR: pons-to-midbrain volume ratio, SCP: superior cerebellar peduncle, MSAc/p: multiple system atrophy-cerebellar/Parkinsonian subtypes, PD: Parkinson disease, PSP: progressive supranuclear palsy) (JPG 123 KB)
