# Supplementary figures and images for: Toward More Accessible Fully Automated 3D Volumetric MRI Decision Trees for the Differential Diagnosis of Multiple System Atrophy, Related Disorders, and Age-Matched Healthy Subjects
Source: Cerebellum. 2022 Sep 26;22(6):1098–108. doi: 10.1007/s12311-022-01472-7 (PMC10657274; doi:10.1007/s12311-022-01472-7)

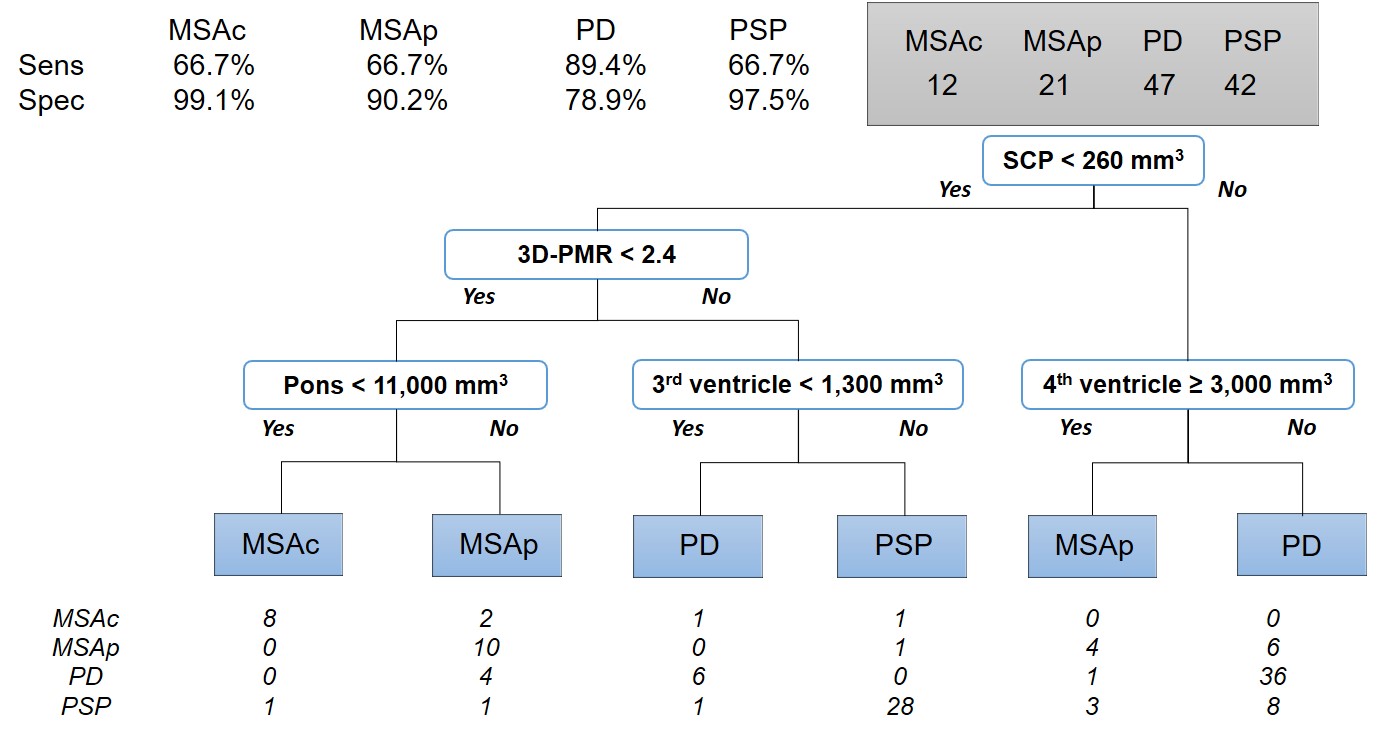

Supplement: Supplementary file 1 — Supplementary Fig.1 Parkinsonism differential diagnosis tree – MSAc vs MSAp vs PD vs PSP who had parkinsonism with or without ataxia at the time of MRI. (3D-PMR: pons-to-midbrain volume ratio, SCP: superior cerebellar peduncle, MSAc/p: multiple system atrophy-cerebellar/Parkinsonian subtypes, PD: Parkinson disease, PSP: progressive supranuclear palsy) (JPG 123 KB) [file 12311_2022_1472_MOESM1_ESM.jpg]
